# Chiral quantum supercrystals with total dissymmetry of optical response

**DOI:** 10.1038/srep23321

**Published:** 2016-03-18

**Authors:** Anvar S. Baimuratov, Yurii K. Gun’ko, Alexander V. Baranov, Anatoly V. Fedorov, Ivan D. Rukhlenko

**Affiliations:** 1 ITMO University, 197101 Saint Petersburg, Russia; 2 School of Chemistry and CRANN Institute, Trinity College, Dublin, Dublin 2, Ireland; 3 Monash University, Clayton Campus, Victoria 3800, Australia

## Abstract

Since chiral nanoparticles are much smaller than the optical wavelength, their enantiomers show little difference in the interaction with circularly polarized light. This scale mismatch makes the enhancement of enantioselectivity in optical excitation of nanoobjects a fundamental challenge in modern nanophotonics. Here we demonstrate that a strong dissymmetry of optical response from achiral nanoobjects can be achieved through their arrangement into chiral superstructures with the length scale comparable to the optical wavelength. This concept is illustrated by the example of the simple helix supercrystal made of semiconductor quantum dots. We show that this supercrystal almost fully absorbs light with one circular polarization and does not absorb the other. The giant circular dichroism of the supercrystal comes from the formation of chiral bright excitons, which are the optically active collective excitations of the entire supercrystal. Owing to the recent advances in assembly and self-organization of nanocrystals in large superparticle structures, the proposed principle of enantioselectivity enhancement has great potential of benefiting various chiral and analytical methods, which are used in biophysics, chemistry, and pharmaceutical science.

Nanostructuring allows the creation of new materials that exhibit unusual physical properties^1^,^2^. Creating and engineering artificial optical activity of nanostructured materials is of fundamental interest and great practical importance due to various applications in photonics, biomedicine, and pharmaceutical industry^3^,^4^,^5^. Such activity is exhibited by arrays or assemblies of chiral nanoobjects with achiral spatial arrangements, and arrays of achiral nanoobjects that are arranged in chiral patterns. The nanoobjects can be plasmonic nanoparticles^6^, nanoparticle pyramids^7^, complexes of gold rods and chiral semiconductor nanocrystals^8^, as well as complexes of gold nanorods and cellulose nanocrystals^9^. Creating, engineering, and dynamically controlling artificial optical activity of nanostructured materials requires further theoretical research and the development of advanced theoretical models of such materials.

If a nanostructured material is made of semiconductor quantum dots (QDs) and the QDs are arranged in a periodic lattice, then it is referred to as a QD supercrystal or a QD superparticle^10^,^11^,^12^,^13^,^14^,^15^. Superparticle structures is an exciting class of anisotropic nanomaterials which possess unique properties and are expected to find a broad range of important applications^12^,^16^. The optical activity of QD supercrystals can originate from the intrinsic optical activity of individual QDs^17^,^18^,^19^ or the chiral arrangement of optically inactive QDs. Both kinds of supercrystals can be fabricated using various techniques such as the Langmuir–Blodgett fabrication^20^,^21^, molecular beam epitaxy (MBE)^22^, nonlithographic formation by anodic membrane template^23^, DNA-assisted formation^24^, self-assembly of colloidal nanocrystals^25^,^26^,^27^,^28^,^29^,^30^, ordering with liquid crystals^31^,^32^, and ion-beam-assisted self-assembly^33^. There are also quite a few methods that can be used for theoretical modeling of QD supercrystals. One of them employs the tight-binding approximation and considers QDs as multi-orbital artificial atoms^34^,^35^,^36^. It was used, in particular, to demonstrate the existence of topological edge states in two-dimensional supercrystals^34^,^35^. The tight-binding approach is best suited for modeling QD supercrystals fabricated using top-down lithography or bottom-up self-assembly approaches, which produce supercrystals with closely spaced and strongly coupled QDs. The coupling of QDs in a supercrystal is weak if the QDs are well separated by organic or inorganic barriers, which are formed during the fabrication process. Such supercrystals can be adequately described by assuming the formation of Frenkel excitons and using the standard method of modeling exciton states in molecular crystals^10^,^37^,^38^. This method has been recently successfully applied to study collective excitations in a two-dimensional ensemble of periodically arranged semiconductor QDs^11^,^13^. It was demonstrated that the multiple degrees of freedom associated with the possibility to arrange QDs in different spatial patterns allows one to engineer the energy spectrum and wave functions of the excitons, and control the linear optical response of the ensemble. Another advantage of QD supercrystals over the ordinary molecular crystals is in their relatively stronger interaction with light due to the fact that the dipole moments of semiconductor nanocrystals (especially those of elongated QDs and nanorods) are larger than the dipole moments of ordinary molecules.

In this paper, for the first time to best of our knowledge, we theoretically study optical activity of a chiral QD supercrystal. The supercrystal is assumed to be an assembly of achiral semiconductor QDs, which are arranged in a helix similarly to the arrangement of molecules in a chiral nematic (cholesteric) liquid crystal^39^. The helical arrangement can be achieved *via* the DNA-assisted self assembly of nanocrystals, as it was demonstrated with plasmonic nanoparticles^6^. We show that the QD-based supercrystal exhibits giant optical activity and almost complete dissymmetry in optical absorption: fully absorbing one type of circularly polarized light and not absorbing the other. The unique optical properties reported here can potentially take place in a solution of weakly interacting helical QD supercrystals, and may prove useful in chiroptical analysis and photonics applications.

## Results

### Theoretical formulation

Consider a supercrystal made of periodically arranged semiconductor nanocrystals with discrete energy spectrum, which will be referred to as quantum dots (QDs). We assume that the wave functions of the low-energy electronic states of the neighboring QDs in the supercrystal do not overlap significantly, and neglect the exchange interaction between the confined charge carriers^11^,^13^. This assumption holds true, in particular, for self-organized colloidal QDs providing high potential barriers for their electrons and holes^40^,^41^. We also use the approach often employed for studying molecular crystals, and take into account only the first dipole-allowed state of QDs with energy Δ*E* = *E*_*e*_ + *E*_*h*_ + *E*_*g*_, where *E*_*e*(*h*)_ is the confinement energy of electron (hole) and *E*_*g*_ is the band gap of bulk semiconductor. It is common knowledge that interband transitions in nanocrystals with low-symmetry crystal lattice or in extended nanoobjects like nanorods and nanowires made of isotropic semiconductors^42^,^43^ can exhibit strong anisotropy. Such nanocrystals only absorb and emit light of certain linear polarizations. To take into account the anisotropy of interband transitions in the optical response of the supercrystal, we consider QDs as artificial anisotropic molecules with oriented dipole moments.

To demonstrate the principle of enantioselectivity enhancement, we restrict our analysis to quasi-one-dimensional QD supercrystals, which are infinite in the *z* direction. The positions of the unit cell in such supercrystals is set up by vectors *na***e**_*z*_, where *n* = 0, ±1, ±2, … and *a* is the superlattice constant. In the general case, the supercrystal has *N* exciton subbands originating from *N* QDs in each unit cell. To calculate the energy spectrum and wave functions of the supercrystal, we describe each QD in a unit cell by two vectors: the radius-vector 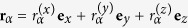 (*α* = 1, 2, …, *N*) of the QD center and the QD dipole moment 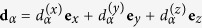. It should be noted that the following analysis is valid regardless of the QD shape, as long as it can be modeled by an oriented point dipole.

**Figure 1:**
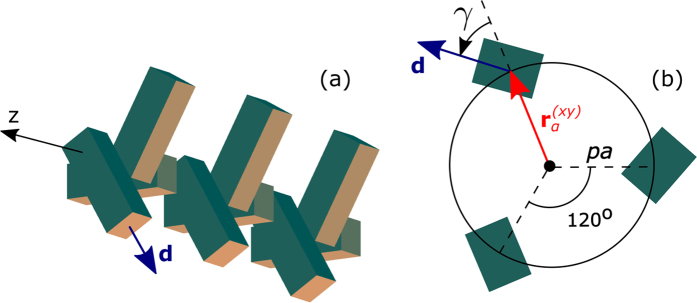
Schematic of a helix supercrystal with three QDs in a unit cell. (**a**) Spatial arrangement of the QDs and (**b**) projection of the unit cell onto the *xy*-plane. QD centers lie on a circle of radius *pa*, where *a* is the superlattice constant, and the helix is parametrically defined by the equations *x*(*t*) = *pa* cos(2*πt*), *y*(*t*) = *pa* sin(2*πt*) and *z*(*t*) = ±*at*, where −∞ ≤ *t* ≤ ∞ and the sign plus or minus corresponds to the right-handed or left-handed helix.

For our supercrystal to exhibit optical activity, it must lack *S*_*n*_ symmetry and be chiral^4^. The chirality can be achieved with at least two QDs in the unit cell, e.g., when the dipole moments of the QDs do not lie in the same plane perpendicular to the supercrystal’s axis. In what follows we focus on a supercrystal in the form of a circular helix shown in Fig. 1(a). We assume that there are three equal QDs in a unit cell, and that the helix pitch is equal to the size of the unit cell *a*. The centers of the QDs lie on the helix, spaced in the *z* direction at *a*/3. The dipole moments of the QDs have the same magnitude, 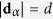, and lie in the *xy*-plane. The angle between vectors **d**_*α*_ and 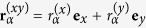, shown in Fig. 1(b) and denoted by *γ* (0 ≤ *γ* < *π*), is assumed to be the same for all QDs. The considered QD supercrystal is described by a set of 18 coordinates shown in Table 1, which depend on four parameters: *a*, *p*, *d*, and *γ*. We show below that these four parameters determine the energies and dispersion of the excitonic subbands and provide much flexibility in tuning the optical activity of the supercrystal.

The energy of excitons can be found using the *Heitler*–*London* approximation through the diagonalization of the resonant interaction matrix^38^


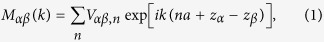


where *k* is the exciton wave number, {*α*, *β*} = {1, 2, 3}, the summation is evaluated over all integers if *α* ≠ *β*, and the term with *n* = 0 is excluded if *α* = *β*. We assume that the QDs are not charged and coupled by the Coulomb potential. Then the matrix element in Eq. (1), calculated in the dipole approximation, is given by


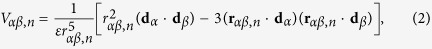


where **r**_*αβ*,*n*_ = **r**_*α*_ − **r**_*β*_ + *na***e**_*z*_ and *ε* is the effective permittivity, which allows for the field screening by the material of QDs and the supercrystal host. Note that the dipole–dipole interaction predominates for closely-packed ensembles of nonspherical QDs in the case of dipole-allowed transitions^44^. Using the parameters of the supercrystal from Table 1 and solving the standard eigenvalue problem, 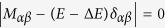, we obtain the following three exciton subbands (see Methods):


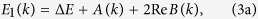



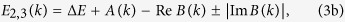


where *A*(*k*) and *B*(*k*) are given in Eqs (12a,b). The interaction between the QDs is seen to remove the degeneracy of the first excited state of the QD superlattice, splitting the corresponding energy level into three exciton subbands. This is *Davydov splitting*, which was initially introduced for molecular crystals with complex unit cells^38^.

**Figure 2:**
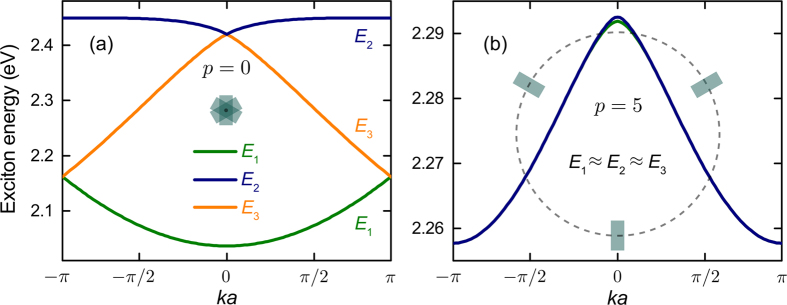
Energy spectrum of excitons supported by two InAs QD supercrystals. The spectra of both supercrystals, with *a* = 6 nm, (**a**) *p* = 0 and (**b**) *p* = 5, is almost independent of *γ* (see Fig. 1). For material parameters see Methods.

Figure 2 shows the exciton energy spectra for a topologically degenerate linear supercrystal, with all QDs located on the *z* axis (*p* = 0), and a true helix supercrystal, whose QDs are at distance 5*a* from its axis. Davydov splitting is pronounced for *p* ~ 1 and reduces with *p*. When the radius of the helix is increased, the subbands merge together as the supercrystal turns into three almost noninteracting linear chains of equally oriented QDs spaced at 3*a*. Hence, the supercrystals of particular interest are those with 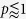. It should also be noted that the energies of the three subbands predominantly depend on material and geometric parameters of the QDs whereas the subbands’ dispersions are controlled by the geometric parameters of the unit cell, scaling as *d*^2^/(*εa*^3^) [see Eq. (12)]. For the parameters of Fig. 2, we have *d*^2^/(*εa*^3^) ≈ 8.2 meV.

**Figure 3:**
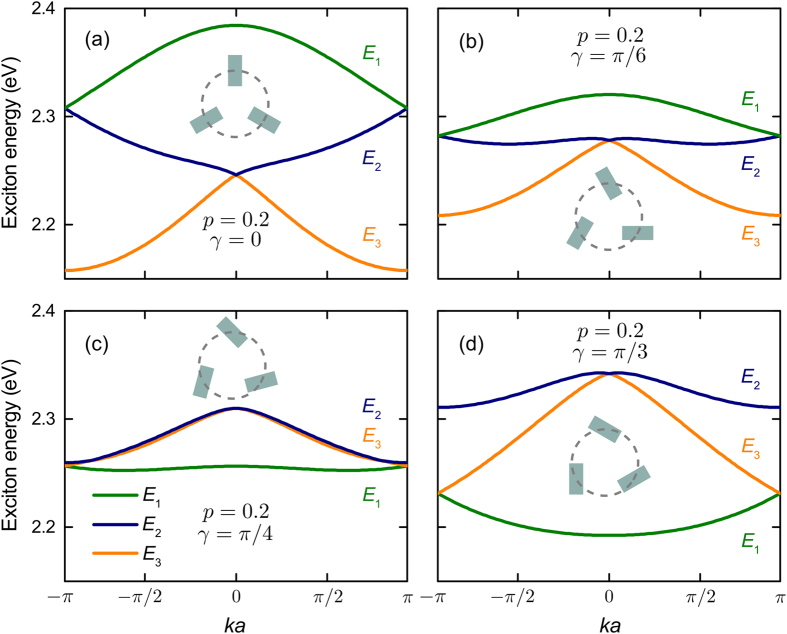
Transformation of exciton energy spectrum with orientations of QDs in a helix supercrystal. Insets show orientations of QDs in a unit cell for (**a**) *γ* = 0, (**b**) *π*/6, (**c**) *π*/4 and (**d**) *π*/3. QDs are made of InAs and the supercrystal parameters are *a* = 6 nm and *p* = 0.2. For material parameters see Methods.

The transformations of the exciton subbands with QD orientations is illustrated by Fig. 3. Three features of the exciton energy spectrum are seen from the figure. First, the variation of *γ* significantly alters the spectrum’s topology, turning the subbands’ minima at points Γ and M into maxima and vice versa. Second, it changes the subbands’ splitting and the position of subband *E*_1_ with respect to subbands *E*_2_ and *E*_3_. As a result, the second and third subbands can become degenerate, as shown in Fig. 3(c) for *γ* = *π*/4. This will lead to the reduction of optical activity of the supercrystal. Third, the dispersions of the second and third subbands at *γ* = 0 are almost linear, which implies massless excitons^45^.

### Circular dichroism and dissymmetry factor

Optical properties of the QD supercrystal are determined by the wave functions of excitons it supports. Owing to the quasi-one-dimensionality of the supercrystal, these wave functions can be written in the form^38^


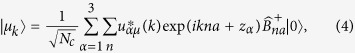


where *N*_*c*_ is the number of unit cells in the supercrystal, *u*_*αμ*_ is the unitary transformation matrix, which diagonalizes the interaction matrix given in Eq. (1), operator 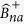 describes transition of the QD positioned at *r*_*α*_ + *na***e**_*z*_ from its ground state to the excited state (the reverse transition is described by 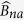), and 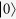 is the ground state of the entire supercrystal. Note that for three QDs in the unit cell, the transformation matrix can be found by analytically solving the 3 × 3 eigenvector problem.

**Figure 4:**
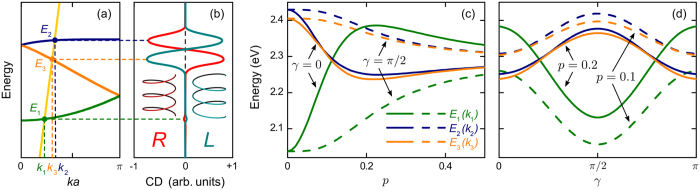
CD spectrum of helix QD supercrystal. (**a**) Energies of three excitons generated by circularly polarized light (yellow is the light line) and (**b**) CD spectra of right-handed (red) and left-handed (teal) supercrystals with three QDs in the unit cell. [(**c**,**d**)] Shifts of the CD peaks with geometric parameters of the supercrystal. All material parameters are the same as in Fig. 3.

The arrangement of QDs with oriented dipole moments in a helix creates a strongly dissymmetric supercrystal, which exhibits a pronounced optical activity. To examine this activity, we calculate the supercrystal’s circular dichroism, defined as the difference between the probabilities of absorption of left-circularly polarized light (LCPL) and right-circularly polarized light (RCPL). The circular dichroism (CD) upon excitation of the *μ*th exciton subband at frequency *ω* is given by


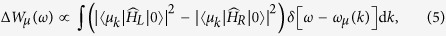


where *ω*_*μ*_(*k*) = *E*_*μ*_(*k*)/*ħ*, and 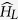 and 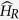 are the Hamiltonians of interaction with LCP and RCP light. If the wave vector of light **q** is directed along the *z* axis, then the two Hamiltonians are of the form


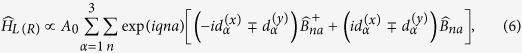


where *A*_0_ is the amplitude of vector potential, 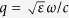, and the upper and lower signs correspond to the LCPL and RCPL, respectively. Using Eqs (4) and (6), we find the matrix elements to be given by


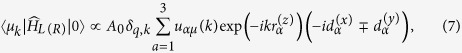


where the Kronecker delta *δ*_*q*,*k*_ takes into account the momentum conservation. According to the energy conservation law, the *μ*th exciton band can only absorb light of wave number *k*_*μ*_ obeying the equation 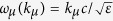. The absorption peaks are centered at energies *E*_*μ*_(*k*_*μ*_), which are shown in Fig. 4(a). The circular dichroism and the dissymmetry factor upon such absorption are given by the expressions


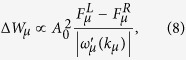



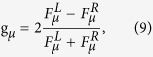


where 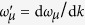 and the transition strengths are defined as


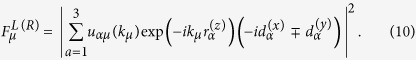


## Discussion

As we have seen in the previous section, the interaction of anisotropic QDs arranged in a chiral supercrystal leads to the formation of excitonic subbands. Owing to the long-range ordering of the QD dipoles in the supercrystals, some of these subbands can exhibit giant optical activity and almost full dissymmetry of optical absorption. Table 2 shows the energies of optical transitions, *E*_*μ*_(*k*_*μ*_), transition strengths, and g-factors for two left-handed QD supercrystals with three QDs in a unit cell. The three exciton subbands are seen to feature significantly different optical properties. The excitons of the first subband hardly absorb any light due to the destructive interference of the dipole moments of QDs in the unit cell, which results in small transition strengths 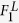 and 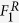. The optical excitation of this subband is almost forbidden, and it is referred to as the subband of *dark excitons*.

Unlike the first subband, the second and third subbands are optically active, representing *bright excitons*. The unique feature of these subbands is that each of them is most efficiently excited with light of one circular polarization. For *p* = 0 the excitons of the high-energy subband 2 predominantly absorb LCPL whereas the excitons of the low-energy subband 3 predominantly absorb RCPL, and the opposite situation occurs for *p* = 0.2. This is evidenced by the dissymmetry factors of bright excitons almost reaching their maximal values of ±2. These excitons remain strongly optically active for a wide range of supercrystal geometries such that the interdot interactions inside a unit cell are comparable to the interactions between the QDs in the adjacent unit cells. This condition ensures the integrity of the supercrystal and is satisfied for *p* ~ 1 regardless of *γ*.

It should be noted that an almost total dissymmetry of the supercrystal’s optical response is a collective phenomenon, which is a consequence of the following four factors: (i) anisotropy of interband transitions in QDs, (ii) arrangement of QDs in the supercrystal with 3_1_ (or 3_2_) screw axis, (iii) perpendicularity of the QD dipoles to the supercrystal’s axis, and (iv) significant Davydov splitting due to the interdot interaction in the unit cell. All these result in matching the scales of material and optical chiralities required for enhanced enantioselectivity in optical response. The absence of any of these factors would diminish the dissymmetry of optical absorption. We have already seen that the Davydov splitting is negligible in supercrystals of large diameters [see Fig. 2(b)]. It is also easy to see that the light propagating along the supercrystal’s axis is not absorbed if all the interband transitions are polarized along this axis. Our analysis also shows that the lowering of the supercrystal’s symmetry due to the noneven spread of QDs along the helix reduces the dissymmetry factors of bright excitons.

The dark and bright excitons manifest themselves in the supercrystal’s absorption spectrum. Figure 4(b) shows the typical CD spectra of two helix supercrystals of opposite handednesses. The positions and strengths of the absorption peaks in the spectra are controlled by the dispersion properties of the exciton subbands and by the difference of the transition strengths, 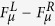. These, in turn, are determined by the material and size of the QDs, entering the energy offset Δ*E* given in Eq. (15), and by the supercrystal geometry—through the coefficients in Eq. (12). Figure 4(c,d) illustrate how the positions of the CD peaks vary with the supercrystal radius and the orientation of the QD dipoles. Due to the finite linewidths of the peaks, the CD signal is the most pronounced for *p* = 0, when splitting 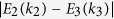 of the bright exciton subbands is maximal. The CD signal from these excitons may vanish in case of accidental degeneracy of the two subbands. Such a degeneracy occurs, in particular, for *p* ≈ 0.07 and *γ* = 0 in Fig. 4(c) and for *p* ≈ 0.2 and *γ* ≈ *π*/2 ± *π*/4 in Fig. 4(d).

In conclusion, we have demonstrated that arrangement of achiral semiconducting nanocrystals in a chiral helix-like assembly results in highly optically active supercrystals. We believe that it will be important to develop this type of quantum superstructures, which may find a wide range of potential applications in chemistry, biotechnology, and photonics.

## Methods

### Exciton energy spectrum

The energy spectrum of excitons supported by our supercrystal is calculated using the *Heitler*–*London* approximation. This approximation is well justified for supercrystals made of semiconductor QDs, because the interdot interaction energy, described by matrix *M*_*αβ*_, is typically much smaller than the excitation energy of the supercrystal. Mathematically, this condition is expressed by the inequality 
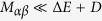
, where *D* is the change in the interaction energy of all QDs constituting the supercrystal with a particular QD induced by the excitation of this QD. Without introducing a significant error, we ignore *D* in all the calculations, as it does not depend on wave number and 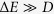. Then the energies of excitons can be represented in the form


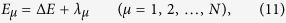


where *λ*_*μ*_ are the eigenvalues of matrix *M*_*αβ*_. The resonant interaction matrices of left-handed (L) and right-handed (R) supercrystals are the complex conjugate of each other, 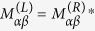. This simply reflects the fact that the supercrystal enantiomers have the same energy spectra but different wave functions, and exhibit different optical activities.

Some algebra shows that the interaction matrix of the left-handed helix QD supercrystal with three QDs in the unit cell, which is described by the parameters in Table 1, is characterized by only two numbers


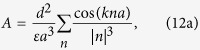



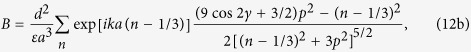


and is given by


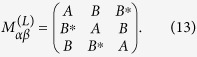


The eigenvalues of this matrix are: *λ*_1_ = *A* + 2 Re *B*, 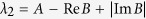, and 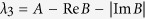.

Figures 2, 3, 4 were plotted using MATLAB software by assuming 32 elementary nodes in all the summations.

### Material parameters

In all the calculations it was assumed that QDs are made of InAs, which is characterized by the following parameters: *m*_*e*_ = 0.023 *m*_0_ (*m*_0_ is the mass of free electron), *m*_*h*_ = 0.41 *m*_0_, *E*_*g*_ = 0.354 eV, *E*_*P*_ = 22.2 eV, and *ε*_QD_ = 12.25. For a simple two-band model of the QD electronic subsystem, these parameters result in the QD dipole moment


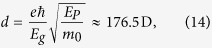


which is equal to 1.765 × 10^−16^ CGS units of dipole moment. For a supercrystal made of spherical QDs of radius *R* = 3 nm, we have


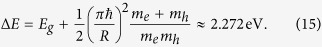


The effective permittivity in Eq. (2) is given by^46^


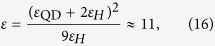


where we have used the host permittivity *ε*_*H*_ = 5.

## Additional Information

**How to cite this article**: Baimuratov, A. S. *et al.* Chiral quantum supercrystals with total dissymmetry of optical response. *Sci. Rep.* **6**, 23321; doi: 10.1038/srep23321 (2016).

## Figures and Tables

**Table 1:** Morphology of helix QD supercrystal shown in Fig. 1.

*α*	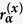	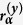	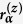	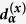	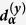	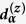
1	−*pa*/2	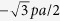	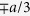	*d* cos(*γ* − 2*π*/3)	*d* sin(*γ* − 2*π*/3)	0
2	*pa*	0	0	*d* cos *γ*	*d* sin *γ*	0
3	−*pa*/2	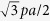	±*a*/3	*d* cos(*γ* + 2*π*/3)	*d* sin(*γ* + 2*π*/3)	0

The upper (lower) sign of 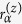 corresponds to right-handed (left-handed) supercrystal.

**Table 2:** Energies and dissymmetry factors of exciton transitions in two left-handed helix QD supercrystals with three QDs in a unit cell.

	*μ*	*E*_*μ*_(*k*_*μ*_) (eV)	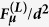	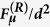	g_*μ*_
*p* = 0	1	2.0373	0.0041	0.0044	0.0756
	2	2.4296	0.0070	2.9866	1.9906
	3	2.4052	2.9866	0.0064	−1.9915
*p* = 0.2	1	2.3837	0.0070	0.0064	0.0945
	3	2.2511	0.0064	2.9866	−1.9915
	2	2.2374	2.9866	0.0070	1.9906

In both cases *γ* = 0 and the supercrystals’ energy spectra are shown in Figs 1(a) and 2(a). The properties of right-handed supercrystals can be found by swapping 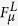 and 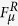 and changing the signs of dissymmetry factors.
